# Single injections of apoA-I acutely improve in vivo glucose tolerance in insulin-resistant mice

**DOI:** 10.1007/s00125-014-3162-7

**Published:** 2014-01-18

**Authors:** Karin G. Stenkula, Maria Lindahl, Jitka Petrlova, Jonathan Dalla-Riva, Olga Göransson, Samuel W. Cushman, Ewa Krupinska, Helena A. Jones, Jens O. Lagerstedt

**Affiliations:** Department of Experimental Medical Science, Lund University, S-221 84 Lund, Sweden

**Keywords:** ApoA-I, Apolipoprotein A-I, Diabetes, Glucose metabolism, HDL, Insulin, Insulin resistance

## Abstract

**Aims/hypothesis:**

Apolipoprotein A-I (apoA-I), the main protein constituent of HDL, has a central role in the reverse cholesterol-transport pathway, which together with the anti-inflammatory properties of apoA-I/HDL provide cardioprotection. Recent findings of direct stimulation of glucose uptake in muscle by apoA-I/HDL suggest that altered apoA-I and HDL functionality may be a contributing factor to the development of diabetes. We have studied the in vivo effects of short treatments with human apoA-I in a high-fat diet fed mouse model. In addition to native apoA-I, we investigated the effects of the cardioprotective Milano variant (Arg173Cys).

**Methods:**

Male C57Bl6 mice on a high-fat diet for 2 weeks that received a single injection of human apoA-I proteins (wild-type and Milano) were analysed for blood glucose and insulin levels during a 3 h incubation followed by glucose tolerance tests. Incorporation of injected human apoA-I protein into HDLs was analysed by native gel electrophoresis.

**Results:**

ApoA-I treatment significantly improved insulin secretion and blood glucose clearance in the glucose tolerance test, with an efficiency exceeding that of lean control animals, and led to decreased basal glucose during the 3 h incubation. Notably, the two apoA-I variants triggered insulin secretion and glucose clearance to the same extent.

**Conclusions/interpretation:**

ApoA-I treatment leads to insulin- and non-insulin-dependent effects on glucose homeostasis. The experimental model of short-term (2 weeks) feeding of a high-fat diet to C57Bl6 mice provides a suitable and time-efficient system to unravel the resulting tissue-specific mechanisms of acute apoA-I treatment that lead to improved glucose homeostasis.

## Introduction

Apolipoprotein A-I (apoA-I), the main protein component of HDL, is cardioprotective and important for reverse cholesterol transport [1]. Both type 2 diabetes and insulin resistance are believed to be associated with reduced HDL functionality and increased risk of cardiovascular disease. Potential roles of apoA-I/HDL functionality in the aetiology of type 2 diabetes with multiple targets for apoA-I/HDL in glucose homeostasis have recently been suggested, including improved insulin secretion [2] and increased glucose uptake in skeletal muscle [3–5] and adipocytes [6]. In addition, infusion of HDL in patients with type 2 diabetes increased both insulin secretion and glucose clearance [4]. While such findings clearly emphasise a connection between HDL function and glucose uptake, an experimental in vivo model is needed to analyse the tissue-specific mechanisms in relation to the integrative effects of apoA-I on whole-body glucose homeostasis.

We investigated the effect of apoA-I on glucose disposal in a model of feeding a high-fat diet (HFD) to mice. Our data show a significant effect of apoA-I wild-type (WT) on glucose-disposal capacity and glucose-dependent insulin secretion after a 3 h treatment. In addition to apoA-I WT, we also analysed the Milano variant (Arg173Cys), of which human carriers display low HDL and increased serum triacylglycerol levels, yet no increase in cardiovascular disease [7], suggesting an atheroprotective function. Whether the apoA-I Milano protein also provides an additionally favourable function on glucose metabolism has not been investigated, and the variant was therefore included in the present study. While the 3 h treatment with apoA-I Milano protein provided similar effects to apoA-I WT on glucose-disposal capacity and glucose-dependent insulin secretion, no additional effects were observed, suggesting that the two proteins are equally efficient.

## Methods

### Expression and purification of recombinant human apoA-I

Human apoA-I containing a His-tag at the N-terminus was expressed in bacteria and purified using immobilised metal affinity chromatography [8] followed by tobacco etch virus protease treatment to remove the His-tag. Protein purity was analysed by SDS-PAGE, and concentration was determined by using a NanoDrop 2000c spectrophotometer (Thermo Scientific, Waltham, MA, USA). Protein function was assayed using a lipid-clearance assay and by qualitative analysis of HDL formation as previously described [8].

### Animals and diets

Male C57BL/6 mice (Taconic, Ry, Denmark) were used at 9-12 weeks of age. Animals were on a 12 h light cycle with non-restricted food and water. The HFD animals were fed an HFD (D12492, 60% fat content; Research Diets, New Brunswick, NJ, USA) for 2 weeks. All animal procedures were approved by the Malmö/Lund Committee for Animal Experiment Ethics, Lund, Sweden.

### Glucose tolerance test

Mice fasted overnight (12 h) were injected i.p. with apoA-I WT or apoA-I Milano (14 mg/kg in *PBS*, pH 7.4; control animals received NaCl) and this was followed by collection of serum samples at the indicated times. In separate experiments, glucose (50 mg/mouse) was injected i.p. 3 h after treatments, followed by collection of serum samples at the indicated times. Blood glucose levels were measured (OnetouchUltra2; Lifescan, Milpitas, CA, USA), and insulin levels were assayed in serum using ELISA (Mercodia, Uppsala, Sweden).

### Immunoblotting

Serum samples from animals 3 h after apoA-I injection were separated by native-PAGE and transferred to nitrocellulose membranes, probed with antibodies to human apoA-I (Abcam No. 64308, Cambridge, UK) and detected with horseradish peroxidase-conjugated (GE Healthcare, Uppsala, Sweden) secondary antibodies. Blots were imaged using the Odyssey Fc system (LI-COR, Lincoln, NE, USA) and analysed using Image studio v2.0 software.

### Statistical analysis

All data are displayed as mean ± SEM. Analysis was performed by one-way ANOVA with Dunnet’s post hoc test or, where indicated, by two-way ANOVA with Bonferroni’s post hoc test or non-parametric Mann–Whitney test using GraphPad Prism software. *p* ≤ 0.05 was considered significant.

## Results

### ApoA-I WT and Milano potentiate glucose-stimulated insulin secretion and increase glucose-disposal capacity

**Fig. 1:**
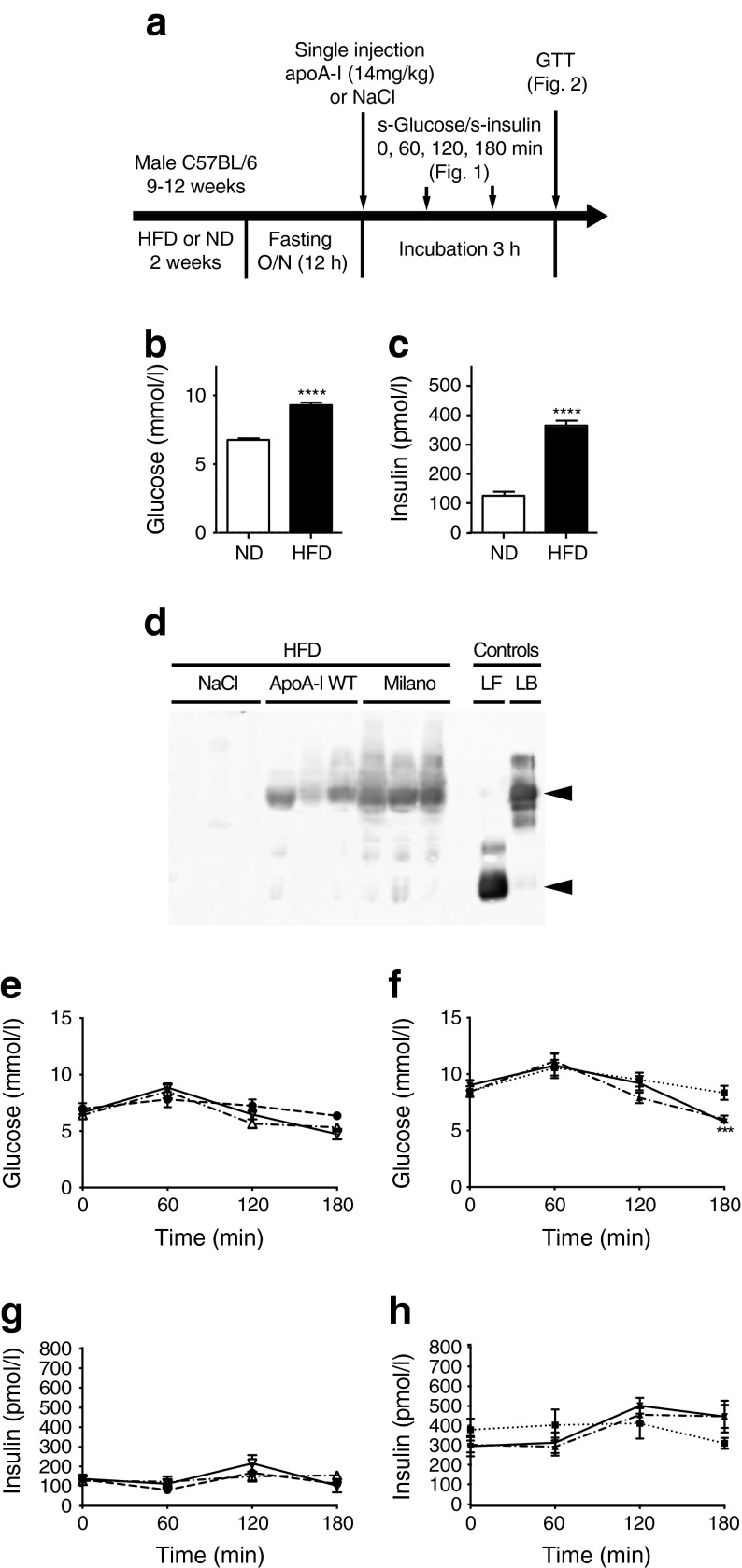
Glucose disposal after acute apoA-I treatment. (**a**) Schematic description of animal treatment and analysis. Mice were analysed for serum insulin (s-insulin) and glucose (s-glucose) after apoA-I injection (this figure) or, in separate groups of mice, for their glucose-disposal capacity in a GTT performed 3 h after apoA-I injection (see Fig. 2). HFD-fed mice exhibited elevated basal fasting glucose (**b**) and insulin (**c**) compared with ND-fed mice. (**d**) Serum samples collected 3 h after injection were separated (2 μl) under native conditions, and the administered human apoA-I was detected using immunoblotting; 0.15 μg purified lipid-free (LF) apoA-I and 0.15 μg synthesised lipid-bound (LB) apoA-I in discoidal HDL particles were used as controls. Arrows indicate migration distance of HDL (top) and lipid-free apoA-I (bottom). Serum glucose (**e**, **f**) and insulin (**g**, **h**) levels at 0, 1, 2 and 3 h after a single injection (14 mg/kg body weight) of apoA-I WT (white triangles/dash-dot line in [**e**, **g**]; black triangles/dash-dot line in [**f**, **h**]), apoA-I Milano (white upside down triangles/solid line in [**e**, **g**]; black upside down triangles/solid line in [**f**, **h**]) or NaCl (black circles/dashed line in [**e**, **g**]; black squares/dotted line in [**f**, **h**]) in fasted ND (**e**, **g**) or HFD (**f**, **h**) animals are shown. (**b**, **c**) *n* = 29; ****p < 0.0001, HFD mice vs ND mice using non-parametric Mann–Whitney test. (**e**–**h**) *n* = 6–8; ***p < 0.001 for apoA-I (WT or Milano) vs NaCl HFD control using two-way ANOVA with Bonferroni’s post hoc test

To investigate the effect of apoA-I WT or Milano on glucose disposal, we used HFD-fed mice (Fig. 1a), which after 2 weeks displayed elevated fasting serum glucose (Fig. 1b) and insulin levels (Fig. 1c). Next, HFD-fed mice (and normal diet [ND]-fed mice as control) were fasted for 12 h followed by a single dose of apoA-I (14 mg/kg i.p.) or an equal volume of NaCl solution. Plasma from apoA-I-treated HFD-fed mice was analysed for formation of lipid–protein complexes (reconstituted HDL) by native gel electrophoresis and immunoblotting using human-specific primary antibodies to apoA-I (plasma from non-treated animals [NaCl control mice] shows no cross-reactivity). As can be seen in Fig. 1d, the i.p. injected apoA-I proteins are bioactive and, at the 3 h time point, have become associated with endogenous lipids and/or lipoprotein particles to form HDL of a size corresponding to that of recombinant discoidal 9.6 nm HDL. We next monitored serum glucose and insulin levels at 0, 60, 120 and 180 min after injection in ND and HFD mice. No significant differences in glucose or insulin levels were observed between the ND-fed groups (Fig. 1e, g). Similarly, no difference in glucose levels was observed between the HFD-fed groups during the first 120 min. However, 180 min after injection, the serum glucose levels in lipoprotein-treated animals were significantly lower than in the HFD control at 180 min (Fig. 1f). In contrast, the insulin levels were not significantly changed during incubation (Fig. 1h).

**Fig. 2:**
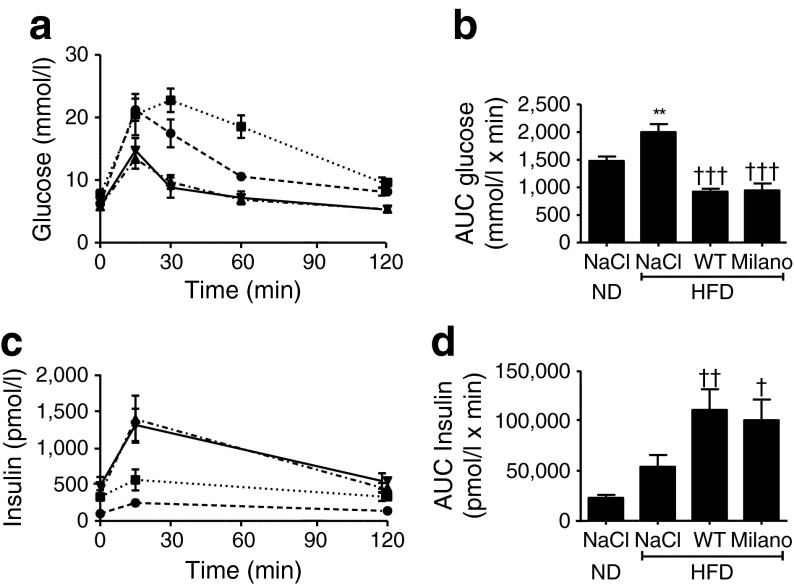
Acute apoA-I treatment improves glucose-disposal capacity in GTTs. ND and HFD mice were treated for 3 h with a single injection (14 mg/kg body weight) of apoA-I WT (triangles/dash-dot line in HFD), apoA-I Milano (upside down triangles/solid line in HFD) or NaCl (circles/dashed line in ND; squares/dotted line in HFD). Mice received an i.p. glucose load (50 mg/mouse) 3 h after injection of NaCl, apoA-I or apoA-I Milano followed by determination of (**a**) glucose and (**b**) insulin concentration at the indicated time points. (**c**) and (**d**) show the AUC values of glucose and insulin levels, respectively, during the GTT. *n* = 6–8; **p < 0.01 for NaCl HFD control vs NaCl ND control; ^†^ *p* < 0.05, ^††^ *p* < 0.01, ^†††^ *p* < 0.001 for apoA-I (WT or Milano) vs NaCl HFD control

Next, glucose tolerance tests (GTTs) were performed on ND and HFD mice (fasted 12 h) that had received a single dose of apoA-I (14 mg/kg) or an equal volume of NaCl solution, 3 h before the test. Mice receiving apoA-I WT or Milano showed a pronounced increase in glucose-clearance capacity (Fig. 2a, b) compared with both HFD and ND mice that had received NaCl, which was accompanied by greatly increased levels of serum insulin (Fig. 2c, d). The effect on insulin secretion was glucose-dependent, since 3 h of apoA-I treatment did not significantly alter insulin levels of the treated animals (Fig. 1e, f). Notably, apoA-I WT and Milano treatment improved both glucose clearance (Fig. 2b) and insulin secretion (Fig. 2d) to a similar extent.

## Discussion

ApoA-I knockout mice exhibit increased basal serum insulin and glucose when fed normal chow diet [5], and adenovirus-mediated apoA-I overexpression in mice has been shown to affect metabolic processes [9]. While such analyses provide important information on long-term effects of changed plasma apoA-I levels, compensatory mechanisms may mask the initial events that lead to an altered metabolic profile. To understand early metabolic changes we analysed the acute effects of apoA-I injection in mice.

We conclude that the elevated apoA-I levels of the treated animals lead to a greatly increased capacity to clear blood glucose, partly associated with improved glucose-stimulated insulin secretion (Fig. 2c, d). Previous in vitro and ex vivo studies suggest that apoA-I treatment also has an insulin-independent glucose-lowering effect, a process in which muscle tissue [3–5] and potentially adipose tissue [6] are likely to play significant roles. The GTT in our study cannot determine whether there are any direct apoA-I effects on the peripheral tissues. However, in line with earlier in vitro and ex vivo findings, we observed reduced glucose levels during the 3 h incubation with apoA-I before the GTT glucose load (Fig. 1f). This suggests pleiotropic functions of apoA-I in integrative glucose metabolism, involving enhanced insulin secretion and consequent improved insulin-dependent glucose uptake, as well as insulin-independent glucose disposal in peripheral tissues. The finding that these effects were similar for the cardioprotective apoA-I Milano variant and apoA-I WT suggests that the Milano variant has no greater effect on glucose disposal in the fasted or glucose-stimulated state than apoA-I WT during acute stimulation. Since the study focused on direct effects of the apoA-I proteins, we cannot exclude the possibility that the Milano variant has long-term beneficial effects on glucose and lipid homeostasis. Finally, while HDL is suggested to stimulate beta cell insulin secretion [2], possibly in an ABCA1- and ABCG1-dependent manner [10], our data suggest that the major effect of apoA-I in vivo is not due to direct stimulation of insulin secretion, but rather priming of the beta cells for glucose-stimulated insulin secretion.

In conclusion, we have shown that injected apoA-I protein leads to increased glucose-disposal capacity, which is due to an increase in glucose-stimulated insulin secretion and probably also increased glucose uptake in peripheral tissues as a direct effect of the apoA-I protein. These effects are observed for both apoA-I WT and Milano. Further analyses are needed to determine the specific mechanisms exerted by the apoA-I protein on lipid and glucose metabolism. Such studies should include comprehensive and integrative analyses of peripheral insulin target tissues (liver, muscle and adipose tissue) and pancreatic islet cells, as well as analysis of alterations in plasma metabolites.

## Abbreviations

apoA-I: Apolipoprotein A-I
GTT: Glucose tolerance test
HFD: High-fat diet
ND: Normal diet
WT: Wild-type

## References

[1] Ansell BJ, Navab M, Hama S (2003). Inflammatory/antiinflammatory properties of high-density lipoprotein distinguish patients from control subjects better than high-density lipoprotein cholesterol levels and are favorably affected by simvastatin treatment. Circulation.

[2] Fryirs MA, Barter PJ, Appavoo M (2010). Effects of high-density lipoproteins on pancreatic beta-cell insulin secretion. Arterioscler Thromb Vasc Biol.

[3] Dalla-Riva J, Stenkula KG, Petrlova J, Lagerstedt JO (2013). Discoidal HDL and apoA-I-derived peptides improve glucose uptake in skeletal muscle. J Lipid Res.

[4] Drew BG, Duffy SJ, Formosa MF (2009). High-density lipoprotein modulates glucose metabolism in patients with type 2 diabetes mellitus. Circulation.

[5] Han R, Lai R, Ding Q (2007). Apolipoprotein A-I stimulates AMP-activated protein kinase and improves glucose metabolism. Diabetologia.

[6] Zhang Q, Zhang Y, Feng H (2011). High density lipoprotein (HDL) promotes glucose uptake in adipocytes and glycogen synthesis in muscle cells. PLoS ONE.

[7] Sirtori CR, Calabresi L, Franceschini G (2001). Cardiovascular status of carriers of the apolipoprotein A-I(Milano) mutant: the Limone sul Garda study. Circulation.

[8] Petrlova J, Duong T, Cochran MC (2012). The fibrillogenic L178H variant of apolipoprotein A-I forms helical fibrils. J Lipid Res.

[9] Van Linthout S, Foryst-Ludwig A, Spillmann F (2010). Impact of HDL on adipose tissue metabolism and adiponectin expression. Atherosclerosis.

[10] Kruit JK, Wijesekara N, Westwell-Roper C (2012). Loss of both ABCA1 and ABCG1 results in increased disturbances in islet sterol homeostasis, inflammation, and impaired beta-cell function. Diabetes.

